# The Influence of Tourists’ Online Value Co-creation Behavior on Consumer-Brand Relationship Quality: The Moderating Effect of Altruism

**DOI:** 10.3389/fpsyg.2022.950546

**Published:** 2022-07-14

**Authors:** Yi Zhang, Yue Liu, Wenxia Tao

**Affiliations:** School of Economics and Management, Shanghai Institute of Technology, Shanghai, China

**Keywords:** “grass planting” marketing, value co-creation behavior, altruism, experience value, consumer-brand relationship quality

## Abstract

In recent years, there is the popular phenomenon of “grass planting” marketing. The value co-creation behavior of ordinary consumers KOC (key opinion consumer) in the online community is sometimes out of utilitarian intentions, which is deemed as plain people’s “grass planting” advertising in a certain degree. We collected the tourists’ data in Chinese Grand Canal National Cultural Park, analyzed the impact of value co-creation behaviors such as tourists’ experience sharing, topic discussions, and suggestions in online communities on the value of tourism experience and the quality of brand relationships under the “planting grass” marketing environment and verified the moderating mechanism of tourist altruism in it. According to the results, tourists’ online value co-creation behavior has a significant positive impact on the consumer-brand relationship quality, and experience value plays a mediating role. Tourists’ online value co-creation behavior has a significant positive impact on experience value, in which altruism plays a moderating role. The greater the tendency of altruism, the higher the impact of tourists’ value co-creation behaviors on their experience value, and vice versa. This conclusion is not only of great significance in deepening and improving theories of value co-creation, altruism, experience value and consumer-brand relationship quality, but also has important certain management enlightenment on how to combine the design of merchant value co-creation incentive mechanism with altruism in “grass planting” marketing.

## Introduction

National Cultural Park is not only a new carrier of a country’s cultural heritage but also an important platform for international cultural exchange. Managers working in the park can take measures to encourage tourists to participate in value co-creation, thus improving the experience value of tourists, as well as customer loyalty (Yi and Gong, 2013). However, according to the latest marketing practice, more and more businesses have been attracting customers to participate in online value co-creation, forming a group of plain consumers KOC (key opinion consumer), while other consumers begin to have some negative comments on them. Under such circumstance, the internal mechanism and boundary of online value co-creation behavior of tourists in National Cultural Park affecting the consumer-brand relationship quality is worthy of further attention from theoretical and practical fields.

Altruism can make subjects carry out behaviors beneficial for the society, and simultaneously enhance their emotional bond, as well as the perceived value (Au et al., 2020; Rong et al., 2021, etc.). Previously, some scholars regarded citizenship behavior as a dimension of value co-creation behavior, which can affect the experience value in combination with participation behaviors (Chan and Kuok, 2021). However, altruism belongs to a concept from the perspective of value, while citizenship behavior belongs to a concept from the perspective of behavior, and there are obvious differences between them. In this paper, taking tourists of the China Grand Canal National Cultural Park as the research object, altruism is introduced into the online value co-creation participation behavior, experience value, as well as the consumer-brand relationship quality model, and the mediating mechanism of experience value and the moderating mechanism of altruism are analyzed. This paper is of great significance for deepening and improving theories of value co-creation, altruism, experience value, and brand relationship quality, and is of enlightening significance on how to combine the design of value co-creation incentive mechanism of park managers with altruism in “grass planting” marketing.

## Theoretical Foundations

### Value Co-creation Behavior

For the first time, Prahalad and Ramaswamy (2010) proposed the concept of value co-creation in 2010; in the process of value creation, both parties jointly build individualized experience through continuous dialog and interaction and jointly determine and solve problems or tasks waiting to be solved. In addition, according to Ramaswamy (2011), in value co-creation, all stakeholders in the whole value chain can be integrated, namely, it can be understood that there are multiple subjects participating in value co-creation. Since the proposition of the value co-creation, the concept of value co-creation behavior has attracted the attention and discussion of many scholars. From a general perspective, scholars generally regard value co-creation behavior as various interactive and cooperative behaviors carried out by participants in value creation (Ranjan and Read, 2016; Shen et al., 2020). In terms of participants in value creation, most scholars generally believe that value co-creation involves not only consumers and enterprises (Ramaswamy, 2011; Grönroos, 2012). Taking canal tourism as the research context, tourists’ online value co-creation behavior is defined as various interactive and cooperative behaviors carried out by tourists to participate in the online value co-creation process.

Based on the connotation of value co-creation, value co-creation behavior can be divided into participation behavior and citizenship behavior (Yi and Gong, 2013). Among these two behaviors, participation behavior refers to the behavior that the subject must carry out to make the value creation succeed. Citizenship behavior refers to the behavior which the subject creates added value for the organization or others independently and voluntarily. In terms of the research on online value co-creation behavior of tourists in this paper, the participation behavior of tourists is taken as the research object and the impact of this behavior on the experience value and the consumer-brand relationship quality will be discussed.

### Altruism

Altruism originated from the field of social psychology and behavioral economics and is defined as “An act aimed at improving interests of others” (Darity et al., 2006), which refers to the behavior and tendency of people to sacrifice their interests for the interests of others in social communication (Penner et al., 2005; Wolfe, 2021), which is not based on the intention of obtaining personal interests, and their roles even exceed requirements of the society on them (Xudong and Ruiyao, 2019).

From the perspective of economics, the essence of altruism is still self-interest (Piccinini and Schulz, 2019; Xiao and Fang, 2019), namely, altruism is also egoism. Although the behavior direction of altruism is an activity that is beneficial to others, it is a benefit in itself, which is not material feedback, but an internal benefit, including the sense of pleasure and achievement obtained after helping others, as well as the kindness and generosity of parents to their children, in which parents’ self-value has also been achieved (Chunyan and Lei, 2015; Azizi et al., 2022). Sometimes this internal benefit will also become the driving force for the subject to continue their behavior (Xudong and Ruiyao, 2019).

Wilson (1976) divided altruism into two varieties, namely, pure altruism (unconditional altruism) and reciprocal altruism (conditional altruism). Relatives are unconditional service objects, while distant relatives and unrelated individuals are conditional service objects. According to the scope of altruistic behavior in the population, altruism can also be divided into two types: kinship altruism and non-kinship altruism. Therefore, it is believed in this paper that altruism among actors is not consistent, namely, these subjects can be divided into those with high initiative in altruistic behavior and those with low initiative in altruistic behavior. For instance, in the behavior of sharing creative ideas, those with high initiative in altruistic behavior have higher perceived value (Jun et al., 2016; Tan et al., 2020). And for staff in the news industry, those with high initiative in altruistic behavior have a higher commitment to their organizations and lower turnover intention than those with low initiative in altruistic behaviors (Xudong and Ruiyao, 2019; Petrakis et al., 2021).

Altruism is to take the initiative to create value for others and act from a higher standing point. Therefore, altruism is introduced into the field of tourism in this paper to explore the impact of tourists’ online value co-creation behavior on their experience value under the action of altruism, based on which canal brand should pay attention to and make full use of altruism, thus obtaining long-term value.

### Experience Value

Experience value (EV) comes into being under the popularity of the experience economy. Although scholars defined experience value from different perspectives, they all have the following three characteristics in common. First of all, it is generally believed that experience value is a subjective feeling of tourists, for example, Kim et al. (2020) and Lin et al. (2020) believed that experience value was the overall feeling of tourists. In addition, it is recognized that experience value is generated in the process of interaction. For instance, Tran et al. (2020) and Cheng H. et al. (2021) believed that experience value embodied characteristics of relativity and interaction. Finally, it is believed that experience value runs through the whole process of tourism activities. Rather (2020) and Peng et al. (2021) believed that experience value could be generated in the whole process of hotel consumption. In brief, it is believed in this paper that experience value refers to the experience and feelings obtained by tourists when interacting with all parties in all links of tourism activities, which may be positive or negative, and it is shared with all parties, with characteristics of value co-creation.

Many scholars applied the theory of customer experience value to the fields of leisure, recreation, and tourism for empirical research, analyzed the factors influencing tourism experience value, and divided these influencing factors into two categories based on the summary of Fang (2013); one category is analyzed from the perspective of tourism experience object, for instance, Haibin (2005) believed that the restaurant environment, theme, and staff’s service and attitude will have a deep influence on customers’ experience value. The other is analyzed from the comprehensive perspective of subjects and objects in tourism. Tourists’ factors (including tourism preferences and expectations, etc.), tourism destination factors (including the product price, etc.), and other factors (including fellow tourists, etc.) will all influence tourists’ experience value (Taoyan, 2009; Deng et al., 2021).

### Consumer-Brand Relationship Quality

Brand relationship quality (BRQ) was first proposed by Fournier (1994) and was defined as the strength and depth of the interaction between consumers and brands, which embodies the strength of the continuous connection between consumers and brands. Fournier (1998) supplemented this definition, he believed that brand relationship quality refers to people’s attitudes and views on the interactive relationship between consumers and brands, which can be simply summarized as the brand relationship quality refers to the strength and depth of the relationship between consumers and brands, which can be used to measure whether the brand relationship is stable and lasting (Fernandes and Moreira, 2019). Referring to Fournier’s definition of consumer-brand relationship quality, this paper takes the consumer-brand relationship quality as the research variable.

To better measure and evaluate the brand relationship quality, existing literature research on the brand relationship quality is sorted out and analyzed, relevant dimensions are shown in Table 1. According to the research on dimensions of brand relationship quality at home and abroad, although there is no clear definition of the dimensions of brand relationship quality in the current academic field, the basic dimensions of brand relationship quality are trust, commitment, as well as loyalty, based on which dimensions of consumer-brand relationship quality is divided into trust, commitment, and loyalty in this paper.

**TABLE 1:** Dimensions of brand relationship quality.

Researcher	Main dimension
Fournier, 1994; Keller, 2001	Love, self-connection, commitment, and intimacy brand partner quality. Loyalty, attachment, commitment, and belonging.
Zhimin and Taihong, 2004	Cognition, emotion, and loyalty
Louis and Lombart, 2010	trust, reliance, and commitment
Nguyen et al., 2016	Interdependence, passion, self-connection, commitment, intimacy, and trust
Barry and Graca, 2019	Trust
Snijders et al., 2020	Trust, honesty, conflict, commitment, and satisfaction
Lan, 2020	Trust, satisfaction, and commitment
Yi, 2020	Satisfaction, trust, and dependence
Wanying et al., 2021	Loyalty

## Research Hypothesis and Conceptual Model

### Research Hypothesis

#### The Influence of Tourists’ Online Value Co-creation Behavior on Experience Value

Many scholars have studied the relationship between value co-creation behavior and experience value. For example, in the context of social commerce, some scholars have analyzed experience value co-creation and its influencing factors, and believe that users’ value co-creation behavior significantly positively affects their experience value (Cheng J. H. et al., 2021; Nadeem et al., 2021). In the context of virtual brand community, some scholars take customers as the core subject of value co-creation, study their behaviors in the value co-creation behavior and its impact, and propose that customers’ value co-creation behavior will affect their experience value (Bu et al., 2020; Siuda, 2020). In the context of commercial banks, some scholars take value co-creation as a research perspective, discuss how value co-creation affects experience value, and give corresponding research strategies (Galdolage and Rasanjalee, 2022). In the context of the automotive service field, some scholars have studied the relationship between value co-creation and service brand experience, and believe that value co-creation has a significant positive impact on service brand and service brand experience (Tchorek et al., 2020). Based on the existing research on value co-creation behavior and experience value in different contexts, this study proposes the following hypothesis based on the context of canal tourism:

H1: Tourists’ online value co-creation behavior positively affects tourist experience value.

#### The Influence of Tourist Experience Value on Consumer-Brand Relationship Quality

For the research on the relationship between experience value and consumer-brand relationship quality, many scholars have analyzed it based on different situations. Based on clothing brand research, some scholars have proposed that experience value in customer perceived value has a direct impact on customer loyalty (Rehman and Shafiq, 2019). Based on the study of shared bicycles, some scholars proposed that experience value significantly positively affects their trust in brands (Durugy et al., 2020; Kim and Kim, 2020), while Jiawei (2021) also concluded that the same is true in the context of virtual knowledge community. Based on the study of virtual brand community, some scholars believe that customer experience value has a significant positive impact on customer satisfaction and customer commitment (Wang et al., 2019; Kumar and Kumar, 2020; Qiao et al., 2021). Many scholars have studied customer perceived value and relationship quality based on the coffee shop industry and proposed that customer perceived value has a positive impact on customer satisfaction, customer trust, and customer commitment (Park et al., 2019; Sethjinda and Laothumthut, 2019; Thamrin et al., 2020). Based on the existing research on experience value and consumer-brand relationship quality in different contexts, this study proposes the following hypotheses based on the canal tourism context:

H2a: Tourist experience value positively affects consumers’ trust to the Canal brand.

H2b: Tourist experience value positively affects consumers’ commitment to the Canal brand.

H2c: Tourist experience value positively affects consumers’ loyalty to the Canal brand.

#### The Moderating Effect of Altruism

In recent years, many scholars have studied altruism. In the field of corporate governance, some scholars have discovered the mediating role of altruism and proposed that altruistic behavior is the embodiment of focusing on corporate relationship governance, and strengthens the emotional connection in the intergenerational inheritance of enterprises (Alnajjar and Hashim, 2020; Miotto and Youn, 2020; Rong et al., 2021). Effective family governance has always been one of the focuses of research in the field of corporate governance. Some scholars believe that in family governance, altruism can build and enhance trust among family members, and have the courage to take responsibility for family businesses (Ruiz-Palomo et al., 2019; Xiao and Fang, 2019). In the field of charitable donation, some scholars believe that altruism has a significant positive impact on individual charitable donation willingness and encourages charitable donation (Gabriel, 2017). Xiaoqing (2019) divided altruism into two dimensions, altruistic personality traits and altruistic values, and came to the same conclusion. We can find that the expressions of the first two scholars both show that altruism can enhance the emotional connection between behavioral subjects. Therefore, in terms of emotional experience, many scholars also put forward their own opinions. For example, Wei et al. (2021) took the crowdsourcing community as the research context, found the moderating effect of altruism, and believed that the behavior of altruists like helping others can bring them satisfaction and pleasure. Qingxing et al. (2019) argued that the pleasure that altruists get from helping others will significantly affect their attitudes, behaviors, and goals when making resource allocation decisions.

Based on the above literature analysis and literature review, we find that:

(1) The research fields of altruism include corporate governance, charitable donation, and so on, so this paper can try to introduce it into the field of tourism.
(2) Altruism is mostly studied as an independent variable, and also has a small part in mediating and moderating effects. This article can try to study it as a moderating variable.
(3) Altruism can enhance perceived value such as the emotional connection between actors, and high altruists have higher perceived value than low altruists.

Therefore, we think: the participation of tourists in value co-creation will increase the brand value of the canal. The reason may be that tourists have invested energy and emotion, and their experience value will be higher, so they will be more loyal to the canal brand; however, this does not consider the level of altruism in value co-creation, so we propose the following hypothesis:

H3: The higher the tendency of altruism, the greater the influence of tourists’ online value co-creation behavior on experience value, and vice versa.

#### The Influence of Tourists’ Online Value Co-creation Behavior on Consumer-Brand Relationship Quality

Many scholars have studied the relationship between value co-creation behavior and consumer-brand relationship quality in different contexts. In the research on social business value co-creation, some scholars believe that there is a significant positive relationship between value co-creation behavior and commitment (Hajli, 2019; Wang et al., 2020). Some scholars also believe that value co-creation has a significant impact on customers’ trust in merchants (Algharabat et al., 2020; Cheng J. H. et al., 2021; Hajli and Shirazi, 2021). However, in the study of virtual brand communities, some scholars believe that the degree of customer participation has a negative moderating effect on the impact of customer self-efficacy on community trust (Yinping, 2017). Based on empirical research in the auto service industry, some scholars have explored how consumers’ participation in value co-creation affects their loyalty (Opata et al., 2021). Similarly, in the study of sports events, some scholars have proposed that multi-value co-creation activities of sports events can have a significant impact on audience value, which cannot only increase their loyalty to the events but also improve their satisfaction (Woratschek et al., 2020; Jiang et al., 2021). Based on the existing research on value co-creation behavior and consumer-brand relationship quality in different contexts, this study proposes the following hypotheses based on the canal tourism context:

H4a: Tourists’ online value co-creation behavior positively affects consumers’ trust to the Canal brand.

H4b: Tourists’ online value co-creation behavior positively affects consumers’ commitment to the Canal brand.

H4c: Tourists’ online value co-creation behavior positively affects consumers’ loyalty to the Canal brand.

### Conceptual Model

Based on the above literature analysis, this paper develops a measure of related constructs in combination with the tourism context of China’s national cultural parks. Among them, tourists’ online value co-creation behavior mainly refers to the concept definition and measurement proposed by Ranjan and Read (2016), Shen et al. (2020), and others. Altruism mainly refers to the concept definition and measurement of Piccinini and Schulz (2019) and Wolfe (2021). The tourist experience value mainly refers to the concept definition and measurement of Kim et al. (2020); Rather (2020), Tran et al. (2020), and Deng et al. (2021). Consumer-brand relationship quality mainly refers to the definition of Fournier (1998); Fernandes and Moreira (2019), and others, and its measurement mainly refers to Barry and Graca (2019), Snijders et al. (2020), and Wanying et al. (2021). Finally, the conceptual model designed in this paper is as follows.

## Methods and Findings

### Pre-investigation

According to the function mechanism model of tourists’ online value co-creation behavior and consumer-brand relationship constructed in this paper, we developed the corresponding questionnaire. To ensure the reliability and validity of the questionnaire, a preliminary survey is hereby conducted. Pre-investigation adopts the way of mutual filling and multi-channel collection. Finally, 62 samples were collected, 5 invalid samples were excluded, and 57 questionnaires were used as the basis of pre-investigation. The analysis of the pre-investigation results shows that the reliability meets the standard (the reliability coefficient of the total table is 0.949 and the coefficient α of each variable is greater than the lowest value of 0.70). In the factor analysis, the factor load of some items failed the test, so it was deleted. After pre-investigation, the final item was determined.

### The Descriptive Statistical Analysis

The questionnaire survey is completed using field distribution, e-mail delivery, the promotion of platform, and so on. A total of 216 questionnaires were issued on February 21, 2022, 32 questionnaires with seriously invalid information were excluded and 184 valid questionnaires were collected, with an effective rate of 85%. The descriptive statistical analysis is shown in Table 2.

**TABLE 2:** The descriptive statistical analysis.

Category	Feature	Frequency	Percentage
Gender	Male	79	43%
	Female	105	57%
Age	18 and under	2	1%
	18–24	36	20%
	25–30	70	38%
	31–40	45	24%
	41–50	29	16%
	51–60	2	1%
Education	Junior high school and below	11	6%
	Senior high school/technical secondary school/technical school	22	12%
	Specialty	73	40%
	Undergraduate course	65	35%
	Master’s degree or above	13	7%
Occupation	Personnel of government departments at all levels, enterprises and institutions, party and government organs and public organizations	23	12%
	Professional and technical personnel (teachers, doctors, engineers, writers, etc.)	12	6%
	Staff (persons engaged in general affairs)	76	41%
	Industrial workers	5	3%
	Housewife	8	4%
	Private enterprise owner	13	7%
	Students	7	4%
	Others	40	22%
Annual household income	1,00,000 and under	32	17%
	1,00,001–2,00,000	73	40%
	2,00,001–3,00,000	62	34%
	3,00,001–4,00,000	13	7%
	4,00,001–5,00,000	3	2%
	Over 5,00,000	1	1%

### Reliability and Validity Analysis of Large Sample Survey Data

In this paper, SPSS26.0 is used to analyze the reliability coefficient (Cronbach’s α). The results show that the reliability coefficient of the total scale is 0.910 and the α coefficients of each variable are greater than the lowest value of 0.7. Therefore, the reliability of the questionnaire is high and it is suitable for validity tests.

Validity analysis usually includes content validity, aggregation validity, and discrimination validity. Since this paper refers to the existing maturity scale, the items and variables meet the requirements of content validity.

After that, the factor analysis of the items shows that the Bartlett approximate chi-square is 2230.311, *P* < 0.001, the KMO index is 0.887, and the data can be analyzed by factor analysis. The results show that the value of CR is more than 0.7, the value of Ave is more than 0.5, and the convergence validity test is tested. The value of *x*^2^/df is 1.528, less than 3, the adaptation is ideal, and the RMSEA value is 0.054, less than 0.1, the adaptation is ideal. The values of GFI, NFI, IFI, TLI, and CFI were more than 0.9, the discriminant validity was passed, and the adaptation effect was good. Taken together, the overall model fits well, and the test data are shown in Table 3.

**TABLE 3:** Reliability and validity test.

Variable	Measuring term	Factor load	α	AVE	CR
Tourists’ online value co-creation behavior (TOV)	TOV1	0.615	0.817	0.526	0.814
	TOV2	0.802			
	TOV3	0.803			
	TOV4	0.661			
Experience value (EV)	EV1	0.763	0.911	0.569	0.913
	EV2	0.662			
	EV3	0.755			
	EV4	0.780			
	EV5	0.641			
	EV6	0.765			
	EV7	0.778			
	EV8	0.868			
Altruism (ALT)	ALT1	0.751	0.905	0.577	0.905
	ALT2	0.711			
	ALT3	0.710			
	ALT4	0.726			
	ALT5	0.853			
	ALT6	0.754			
	ALT7	0.767			
Trust (T)	T1	0.746	0.866	0.614	0.864
	T2	0.750			
	T3	0.831			
	T4	0.805			
Commitment (C)	C1	0.776	0.769	0.634	0.776
	C2	0.816			
Loyalty (L)	L1	0.724	0.788	0.686	0.812
	L2	0.921			

*x^2^/df = 1.528, RMSEA = 0.054, GFI = 0.901, NFI = 0.903, IFI = 0.964, TLI = 0.954, CFI = 0.963.*

### Correlation Analysis

The correlation between the variables is tested, and the results are shown in Table 4. There is a significant correlation between tourists’ online value co-creation behavior, experience value, trust, commitment, and loyalty.

**TABLE 4:** Decribes the results of statistics and correlation analysis.

Variable	*M*	*SD*	1	2	3	4	5	6
Tourists’ online value co-creation behavior	3.45	0.73	0.725					
Experience value	4.04	0.63	0.203**	0.754				
Altruism	4.14	0.69	0.344**	0.501**	0.760			
Trust	4.05	0.61	0.214**	0.612**	0.544**	0.784		
Commitment	3.94	0.55	0.254**	0.627**	0.657**	0.548**	0.796	
Loyalty	3.96	0.84	0.300**	0.566**	0.559**	0.549**	0.603**	0.828

At level 0.05 (double tail), the correlation was significant. Diagonal value is the square root of AVE. **At 0.01 level (double tail), the correlation was significant. Sex: 1 = male, 2 = female; age range: ① *18 and below*, ② *18–24 years old*, ③ *25–30 years old*, ④ *31–40 years old*, ⑤*41–50 years old, and* ⑥ *51–60 years old.*

### Hypothesis Test

#### The Relationship Between Tourists’ Online Value Co-creation Behavior and Trust: The Mediation Effect of Experience Value

First of all, we used Model 4 (Model 4 is a simple intermediary effect model) in SPSS macro compiled by Hayes (2012) to study the mediation effect of experience value between tourists’ online value co-creation behavior and trust under the control of gender, age, and so on. The result shows that tourists’ online value co-creation behavior plays a significant role in predicting trust (*B* = 0.1933, *t* = 3.1809, *P* < 0.01), H4a is verified.

After adding mediating variables, tourists’ online value co-creation behavior had no significant predictive effect on trust (*B* = 0.0893, *t* = 1.7832, *p* > 0.05), but tourists’ online value co-creation behavior had a significant positive effect on experiential value (B = 1.1824, *t* = 2.8735, *p* < 0.01), and experience value had a significant positive effect on trust (*B* = 0.5706, *t* = 9.9274, *p* < 0.001). This shows that experience value plays a complete intermediary role between tourists’ online value co-creation behavior and trust. H1 and H2a are tested.

#### The Relationship Between Tourists’ Online Value Co-creation Behavior and Commitment: The Mediation Effect of Experience Value

After that, this paper uses the same method to test the mediation effect of experience value between tourists’ online value co-creation behavior and commitment under the control of gender and age. The result shows that tourists’ online value co-creation behavior plays a significant role in predicting commitment (*B* = 0.1984, *t* = 3.656, *p* < 0.001), H4b is verified.

After putting in the mediating variable, the predictive effect of tourists’ online value co-creation behavior on commitment was still significant (*B* = 0.1984, *t* = 3.656, *p* < 0.001), the positive effect of tourists’ online value co-creation behavior on experience value was significant (*B* = 1.1824, *t* = 2.8735, *p* < 0.01), and the positive effect of experience value on commitment was significant (*B* = 0.523, *t* = 10.3477, *p* < 0.001). This shows that experience value plays a mediating role between tourists’ online value co-creation behavior and commitment. In addition, the upper and lower limits of the Bootstrap 95% confidence interval of the direct effect of online value co-creation on commitment and the mediating effect of experience value do not contain 0, as shown in Figure 3. It shows that tourists’ online value co-creation behavior can not only predict commitment directly but also predict commitment through the intermediary role of experiential value. The direct effect (0.103) and the mediating effect (0.0954) account for 52% and 48% of the total effect (0.1984), respectively. H2b is tested.

**FIGURE 1:**
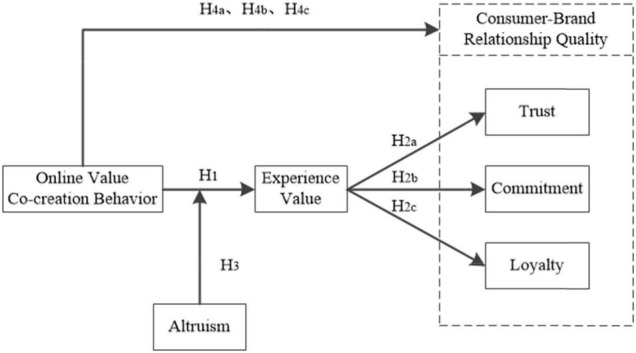
The model of this research.

**FIGURE 2:**
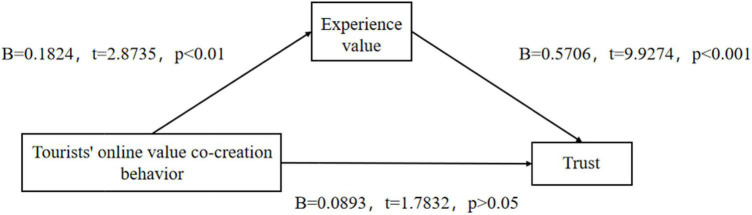
Test of mediating effect of experiential value-1.

**FIGURE 3:**
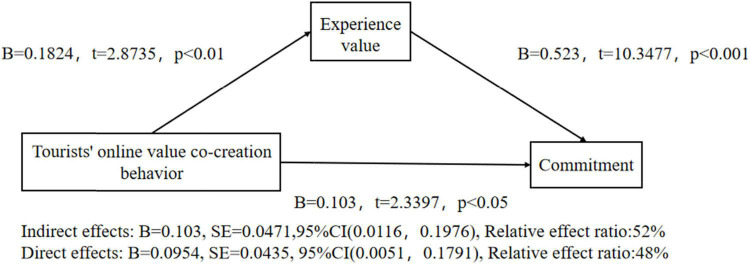
Test of mediation effect of experiential value-2.

#### The Relationship Between Tourists’ Online Value Co-creation and Loyalty: The Mediation Effect of Experience Value

Finally, this paper uses the same method to study the mediation effect of experience value between tourists’ online value co-creation behavior and loyalty under the control of gender and age. The result shows that tourists’ online value co-creation behavior plays a significant role in predicting loyalty (*B* = 0.3508, *t* = 4.2185, *P* < 0.001), H4c is verified.

**FIGURE 4:**
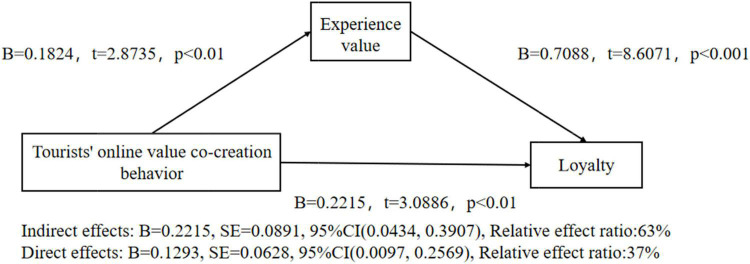
Test of mediation effect of experiential value-3.

After putting in the mediating variable, the predictive effect of tourists’ online value co-creation behavior on loyalty is still significant (*B* = 0.1984, *t* = 3.656, *p* < 0.001), the positive effect of tourists’ online value co-creation behavior on experience value is significant (*B* = 1.1824, *t* = 2.8735, *p* < 0.01), and the positive effect of experience value on loyalty is significant (*B* = 0.523, *t* = 10.3477, *p* < 0.001). This shows that experience value plays a mediating role between tourists’ online value co-creation behavior and loyalty. In addition, the upper and lower limits of the Bootstrap 95% confidence interval of the direct effect of online value co-creation on loyalty and the mediation effect of experience value do not contain 0, as shown in Figure 4. It shows that tourists’ online value co-creation behavior can not only directly predict loyalty but also predict loyalty through the mediating role of experience value. The direct effect (0.2215) and the intermediary effect (0.1293) account for 37% and 63% of the total effect (0.3508), respectively. H2c is tested.

#### The Relationship Between Tourists’ Online Value Co-creation Behavior and Experience Value: The Moderating Effect of Altruism

Because altruism only plays a moderating effect between online tourists’ value co-creation behavior and experience value, and the outcome variables in the model include three dimensions, this paper uses model 1 (model 1 is used to verify the simple moderating effect) in the SPSS macro compiled by Hayes (2012) to test it under the control of gender and age. The results are shown in Table 5. After altruism is put into the model, the product of tourists’ online value co-creation behavior and altruism has a significant predictive effect on experience value (*b* = 0.1124, *t* = 2.0055, *P* < 0.05); H3 is true.

**TABLE 5:** Moderating effect model test.

Predictive variable	Result variable: experience value	
	*B*	*t*
Constant	4.1798	80.1861***
Tourists’ online value co-creation behavior	0.1678	2.092*
Altruism	0.21	2.5834**
Tourists’ online value co-creation behavior × altruism	0.1124	2.0055*
*R*-sq	0.2786***	
*F*	11.9313***	

***P < 0.001, **P < 0.01, and *P < 0.05.

**FIGURE 5:**
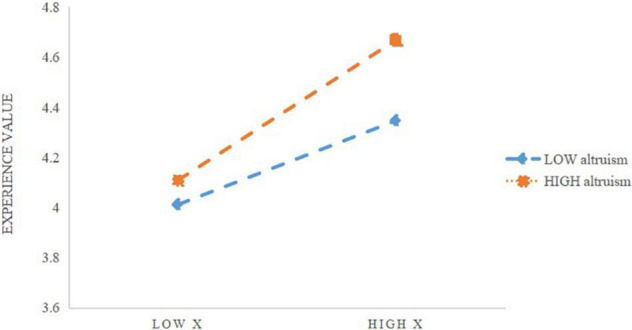
Simple slope analysis of altruism.

To clarify the essence of the moderating effect of altruism, a simple slope analysis is further carried out. The results are shown in Figure 5.

## Conclusion and Recommendations

### Summary of Findings

Based on the survey data of the Grand Canal National Cultural Park, this paper explores the relationship between tourists’ online value co-creation behavior and consumer-brand relationship quality and verifies the mediating effect of experience value and the moderating effect of altruism. The research shows that online value co-creation behavior significantly and positively affects the sub-dimensions of experience value and consumer-brand relationship quality, namely trust, commitment, and loyalty. Experience value positively affects tourists’ trust, commitment, and loyalty to the canal brand. Altruism plays a moderating role between co-creation behavior and brand relationship quality, and the online value co-creation behavior of high altruists has a higher coefficient of influence on experience value, and vice versa. Experience value plays a mediating role between co-creation behavior and brand relationship quality, and it also plays a complete mediating effect between online value co-creation behavior and trust, that is, after adding the variable of experience value, the effect between online value co-creation behavior and trust is fully realized through experience value. Experience value plays a partial mediating effect between online value co-creation behavior and commitment, online value co-creation behavior and loyalty, the relative effects accounted for 48 and 37%, respectively. This shows that the mediating effect of experience value is not equal, and when the outcome variables are different, the mediating effect is also different. This further suggests that the utility of experience value between online value co-creation behavior and loyalty, commitment, and trust is gradually increasing.

The theoretical contributions of this study mainly include the following two points: first, in the past, altruism was mainly used as an independent or mediating variable to influence other behavioral variables. Some scholars also regarded citizenship behavior as a dimension of value co-creation behavior, and together with participation behavior as an independent variable to affect an individual’s perceived value. This paper incorporates altruism into the research model of “value co-creation-experience value-brand relationship quality,” and explores its adjustment mechanism. The research results show that the higher the altruistic tendency, the greater the impact of online value co-creation on experience value, and vice versa, this finding enriches and deepens the basic theory of altruism and value co-creation behavior. Second, the construction of China’s national cultural parks is in full swing, but the theoretical research mainly focuses on conceptual analysis, connotation mining, policy recommendations, and so on. The quantitative analysis of the main effect, mediating effect, and moderating effect in this paper is of great value to the theoretical exploration and management practice of China’s national cultural parks.

### Managerial Implications

Tourists’ online value co-creation behavior will be beneficial for the experience value and the sub-dimension of consumer-brand relationship quality, which is more significant among high altruists. Based on the after-effect of online value co-creation behavior, the following reference suggestions are proposed in this paper.

First of all, build a platform to encourage virtual community interaction and promote the systematic establishment of tourism information in cities along the line. Through an open and fair online communication platform, expand channels for tourists to obtain and share information and communicate with the staff of the scenic spot and other tourists, to improve their sense of participation. In virtual communities (Weibo, etc.), tourists can share their tourism experience and opinions, and scenic spots can also encourage tourists to shoot a series of works through new media (micro movies, etc.) and select the best among these works, which furthermore, give timely and effective rewards to tourists’ participation behavior, to make tourists more enthusiastic about participating in these activities. Smart cities and smart scenic spots can be realized through the 5G technology, and a complete and unified tourism website system of cities along the line can be built, to realize online multi-functional services including tourism online service, online marketing, online reservation and payment, expand the amount of information, improve the efficiency of online service, strengthen the publicity through a variety of media, and finally make the canal brand more influential.

Second, insist on altruism to arouse the initiative of tourists. Considering the moderating function of altruism, scenic spot managers must consider tourists’ altruism, formulate corresponding management methods and strategies, and provide corresponding platforms and ways for tourists to value co-creation behaviors. The canal brand can explore the main preferences and demands of tourists in tourism on the corresponding platform according to the requirements of the brand, to provide tourists with a high-quality experience through innovative technologies and tools, fully cultivate the subjectivity and initiative of tourists, and promote the development of the canal brand from the innovative perspective.

## Limitations and Further Research

### Limitations

(1) The scale used in this paper has certain limitations. First of all, for the scale of value co-creation behavior, there is currently no scale developed in the tourism context. This paper only draws on the scale widely used in the service industry and optimizes it based on the canal tourism context. Therefore, the scale still needs to be improved. Second, for the scale of experience value, this paper mainly draws on the scales used by foreign scholars to measure the experience value of domestic tourists in the context of ecotourism, so the results may have certain errors.
(2) Due to time constraints, it is difficult to carry out point-to-point interviews and fill in all interviewees. During the filling process, a small number of respondents appeared to answer the first part of the questions carefully, while the latter part lacked thinking. Therefore, the universality and reliability of the research conclusions will be affected to a certain extent.

### Directions for Future Research

Based on the limitations of this study in terms of research scales and questionnaires, future research can develop higher-quality scales for national cultural parks. Under the support of sufficient conditions, we will further select suitable interviewees and strive to obtain sample data from all provinces across the country to support empirical research, thereby improving the reliability of the empirical results.

In this research, in the “grass planting” marketing environment and the questioning voice of other consumers, the influence boundary of tourists’ value co-creation behavior on experience value in the “grass planting” marketing environment, namely, the moderating mechanism of altruism is explored, and in future research, other moderating variables can be explored by scholars. Furthermore, if consumers question the value co-creation behavior of ordinary consumers KOC (Key Opinion Consumer) in the online community, how do they change their information search model? Finally, how do the altruistic tendencies of ordinary opinion leaders affect the consumption decisions of other consumers?

## Data Availability Statement

The raw data supporting the conclusions of this article will be made available by the authors, without undue reservation, to any qualified researcher.

## Author Contributions

YZ was responsible for the theme determination, participated in the revision of the writing, and contributed to the elaboration of ideas, additional analysis, and final draft. YL participated in determining the theme, was responsible for literature review and initial writing, and then revised the article. WT was involved in determining the theme, analyzing the data, and then revising the article. All authors contributed to the article and approved the submitted version.

## Conflict of Interest

The authors declare that the research was conducted in the absence of any commercial or financial relationships that could be construed as a potential conflict of interest.

## Publisher’s Note

All claims expressed in this article are solely those of the authors and do not necessarily represent those of their affiliated organizations, or those of the publisher, the editors and the reviewers. Any product that may be evaluated in this article, or claim that may be made by its manufacturer, is not guaranteed or endorsed by the publisher.
